# Anatomical Variables of the Superior Thyroid Artery on Computed Tomography Angiograms

**DOI:** 10.3390/medicina61050775

**Published:** 2025-04-22

**Authors:** Rodica Narcisa Calotă, Mugurel Constantin Rusu, Marius Ioan Rusu, Cătălin Constantin Dumitru, Alexandra Diana Vrapciu

**Affiliations:** 1 Division of Anatomy, Faculty of Dentistry, “Carol Davila” University of Medicine and Pharmacy, 020021 Bucharest, Romania; rodica-narcisa.plai@drd.umfcd.ro (R.N.C.); catalin-constantin.dumitru@drd.umfcd.ro (C.C.D.); alexandra.vrapciu@umfcd.ro (A.D.V.); 2 Division of Economic Informatics, Faculty of Cybernetics, Statistics and Economic Informatics, University of Economic Studies, 010374 Bucharest, Romania; rusumarius21@stud.ase.ro; 3 University Emergency Hospital Bucharest, 050098 Bucharest, Romania

**Keywords:** carotid artery, hyoid bone, anatomical variation, thyroid artery, thyroid gland

## Abstract

*Background and Objectives*: The superior thyroid artery (STA) typically has its origin inferiorly to the greater hyoid horn (GHHB) either from the external carotid artery (ECA) or from the carotid bifurcation (CB) or the common carotid artery (CCA). We aimed to determine the topographic variants of the STA related to the GHHB and the artery of origin and to check their bilateral symmetry. *Materials and Methods:* Determinations were performed in a sample of 85 archived angio CT adult cases, comprising 53 men and 32 women. The origins of the STAs from the CCA/CB/ECA were classified as types A–C. We defined the vertical topographies of the STA as follows in relation to the GHHB: type 1 (infrahyoid), type 2 (hyoid), and type 3 (suprahyoid). Subtypes of the STA course were added: “a”, lateral to the GHHB; “b”, medial; and “c”, posterior to it. Unilateral combinations of types and bilateral associations of these were established. *Results:* In 170 carotid axes, we detected STA type A in 8.82%, type B in 28.82%, and type C in 60% of cases. It was absent in 2.35% of the cases. The infrahyoid type 1 of STA was found in 47.06% of cases. The hyoid type 2 was found in 20.59% (2a), 0.59% (2b), and 4.71% (2c). The suprahyoid type 3 was found in 21.18% (3a), 0% (3b), and 3.53% (3c). Thirteen unilateral combinations of types were found. The most prevalent ones were C1 (27.71%), C3a (17.47%), and B1 (15.66%). We established thirty-seven bilateral associations of unilateral combinations of types. The cases with asymmetrical bilateral associations of unilateral combinations of types prevailed. A lowered hyoid bone overlapping the thyroid cartilage was found in one of these cases. The prevailing associations were C1-C1 (bilateral infrahyoid origin of the STA from the ECA, 13/85, 15.29%), C3a-C3a (suprahyoid origins of the STAs from the ECAs and lateral courses over the GHHB, 9/85, 10.58%) and C1-B1 (infrahyoid origins from the ECA and CB, 8/85, 9.41%). *Conclusions:* The vertical topography of the STA is highly variable and hardly predictable but can be examined in imaging studies. The GHHB may be of use to identify and manage the artery. The STA is rarely absent.

## 1. Introduction

The external (ECA) and internal (ICA) carotid arteries originate from the common carotid artery (CCA), commonly at the level of the superior margin of the thyroid cartilage, or at the level of the hyoid bone, or between them [1,2,3,4,5,6]. The carotid bifurcation (CB) is usually located at two-fifths of the distance from the mastoid tip to the sternal extremity of the clavicle and closer to the mastoid tip [7]. The ECA is a major supplier of the head and neck by its branches, the superior thyroid (STA), lingual, facial, occipital, ascending pharyngeal, posterior auricular, superficial temporal, and maxillary arteries. The unknown variations in the origin of ECA and its branching pattern are one of the leading causes of mortality and morbidity during surgery [8].

The thyroid arteries are the major blood suppliers of the thyroid gland. The STA is typically a collateral branch of the external carotid artery (ECA). It may form different common trunks of origin with the neighbouring branches of the ECA, such as the thyrolingual (TLT) or thyrolinguofacial (TLFT) trunks [9,10]. Additionally, the level of origin of the STA from the ECA was determined in original studies [11,12], reported in single cases [13,14,15], and a recent meta-analysis on these aspects was also published [16]. Numerous studies have observed the relevant relationships between the STA and the superior laryngeal nerve [17].

However, the variable topography of the STA, which is related to the greater horn of the hyoid bone (GHHB), appears to have been overlooked. Recent studies have found that the carotid arteries exhibit variable courses and that the topography of the carotid bifurcation (CB) is also variable [18]. Higher levels of the CB can be expected rather often [3]. Therefore, the STA-hyoid topography may also vary. We decided to conduct a specific anatomical investigation to determine the topographical possibilities of the STA course in relation to the GHHB and the bilateral symmetry or asymmetry of the variants.

## 2. Materials and Methods

### 2.1. Study Design

Determinations were performed in a sample of 85 archived angio-CT adult cases, including 53 men and 32 women, with a mean age of 68 ± 12 years. The scans were acquired between June and December 2024. The study was conducted at a tertiary academic medical centre in Bucharest, Romania. The research followed the principles of the World Medical Association Code of Ethics (Declaration of Helsinki). The responsible authorities (affiliation 3) approved the study (approval no. 10540/16 February 2022).

### 2.2. Eligibility Criteria

Inclusion criteria were as follows: (1) high-quality angio-CT scans with clear visualisation of anatomical structures; (2) complete opacification of the carotid arteries and their branches with contrast agent; (3) scans covering both the head and neck regions; and (4) absence of pathological conditions that could distort the carotid anatomy.

Exclusion criteria included the following: (1) poor image quality insufficient for detailed anatomical assessment; (2) incomplete contrast filling of the carotid arteries; (3) presence of pathological processes affecting the cervical vasculature or surrounding structures; and (4) history of surgical intervention in the neck region.

The angio-CT scans were initially acquired for clinical indications unrelated to thyroid, neck, or cervical vascular pathology; they were most commonly used for evaluating or following cerebral vascular conditions. No cases were excluded.

### 2.3. Imaging Protocol

The CT scans were performed as previously with a 32-slice scanner (Siemens Multislice Perspective Scanner, Forcheim, Germany). We used the Horos for macOS (ver.3.3.6, Horos Project, Annapolis, MD, USA) programme, as in previous studies. Findings were checked on two-dimensional reconstructions and three-dimensional volume renderings.

All angio-CT scans included in the study were acquired in the orthostatic position following standard imaging protocols. Prior to assessment, each scan was aligned to ensure consistency and eliminate positional bias. Specifically, alignment was performed by adjusting the dataset so that the median sagittal plane of the scan corresponded precisely with the software’s sagittal guide axis. To verify and correct any horizontal tilt, the zygomatic arches were aligned on the same axial plane, thereby ensuring bilateral symmetry.

### 2.4. Data Collection and Analysis

Two reviewers (R.N.C. and M.C.R.) independently assessed the angio-CT scans. In cases where discrepancies arose between the two assessments, the evaluation provided by the more experienced reviewer (M.C.R.) was considered definitive. To minimise bias, both reviewers were blinded to patient data and assessed the scans using the same standardised alignment and classification protocol described below.

Several anatomical variables of the STA were documented. For the STA origins, types were defined as follows: A—the CCA origin; B—the CB origin; C—the ECA origin; D—the ICA origin; E—TLT; and F—TLFT or other rare morphologies. Regarding the topography and course of the STA, we classified its origin according to the GHHB: type 1—inferior to the GHHB; type 2—at the level of the GHHB (with course subtypes: 2a—lateral to the GHHB; 2b—medial to the GHHB; and 2c—posterior to the GHHB); and type 3—superior to the GHHB (with course subtypes: 3a—lateral to the GHHB; 3b—medial to the GHHB; and 3c—posterior to the GHHB) (Table A1).

Unilateral combination types were recorded (Table A2), and bilateral associations of the types’ combinations were established.

The Microsoft Excel programme was used for the initial analysis to gain a general outlook on the data due to the ease of use for the compiled data format. All subsequent statistical analyses using simple and multiple regression tests and an ANOVA were conducted using the EViews12 statistical programme. Continuous variables across groups were compared (gender, sides, STA origins, and STA courses). Regression was used to identify relationships between these variables and the influences between. A *p*-value of <0.05 was considered statistically significant.

## 3. Results

### 3.1. The Individual Variables of the STA

**Figure 1:**
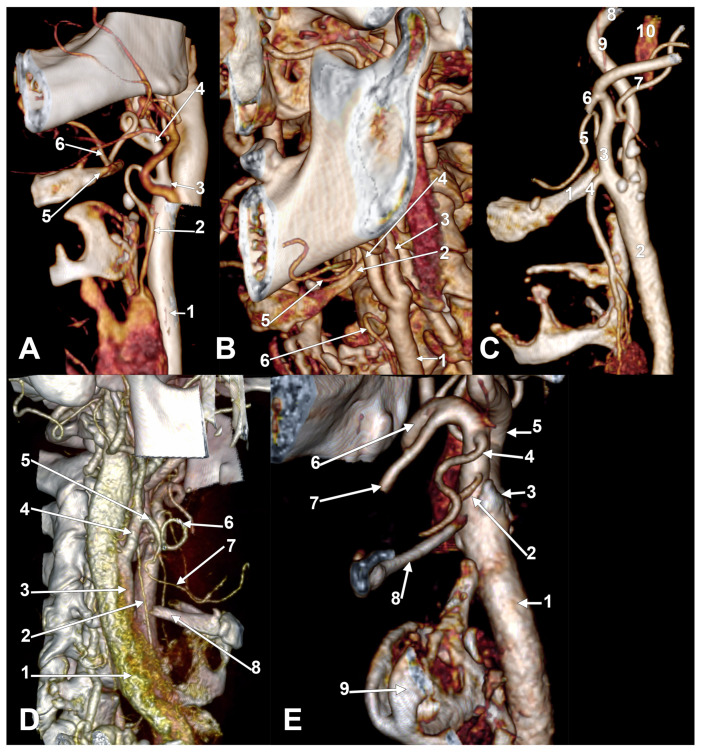
(**A**) Superior thyroid artery originating from carotid bifurcation. Three-dimensional volume rendering. Left side. Female case. Infero-lateral view. 1. Common carotid artery; 2. superior thyroid artery; 3. carotid bifurcation; 4. external carotid artery; 5. greater hyoid horn; 6. lingual artery. (**B**) Superior thyroid artery originating from common carotid artery. Three-dimensional volume rendering. Left side. Female case. Antero-lateral view. 1. Common carotid artery; 2. greater hyoid horn; 3. internal carotid artery; 4. external carotid artery; 5. lingual artery; 6. superior thyroid artery. (**C**) Left superior thyroid artery originating from external carotid artery lateral to greater hyoid horn (unilateral combination C2a). Male case. Lateral view. 1. Greater horn of hyoid bone; 2. common carotid artery; 3. external carotid artery; 4. superior thyroid artery; 5. lingual artery; 6. facial artery; 7. occipital artery; 8. internal carotid artery; 9. ascending pharyngeal artery; 10. internal jugular vein. (**D**) Right superior thyroid artery with suprahyoid and subgonial origin from external carotid artery (1.81 cm long suprahyoid segment, unilateral C3a combination). Male case. Three-dimensional volume rendering. Right infero-lateral view. 1. Internal jugular vein; 2. superior thyroid artery; 3. right common carotid artery; 4. external carotid artery; 5. linguofacial trunk; 6. facial artery; 7. lingual artery; 8. greater hyoid horn. (**E**) Right superior thyroid artery with suprahyoid origin from internal wall of external carotid artery but with lateral course on greater hyoid horn (unilateral combination C3a). Male case. Three-dimensional volume rendering. Antero-medial view. 1. Common carotid artery; 2. superior thyroid artery; 3. carotid bifurcation; 4. lingual artery; 5. internal carotid artery; 6. external carotid artery; 7. facial artery; 8. greater hyoid horn; 9. thyroid cartilage.

In the overall group of 170 carotid axes, we detected the STA origin from the CB in 28.82% (Figure 1A), the CCA in 8.82% (Figure 1B), and the ECA in 60%. The STA was absent in 2.35% of this group. In males (N_M_ = 106 lateral sides), these prevalences were 8.49%/35.85%/52.83%/2.83%. In females (N_F_ = 64 lateral sides), these prevalences were 9.38%/17.19%/71.88%/1.56%. We did not identify any cases with STA origin from the ICA, TLT, or TLFT (types D–F). We did not record any other rare morphologies (Table A3). The A–C types of STAs were classified by side and gender (Table A4).

We determined the bilateral symmetry or asymmetry of the A–C types of STA. Bilateral symmetry of origin from the CCA (type A) was recorded in 1/85 cases (1.18%). Bilateral symmetry of origin from the CB (type B) was identified in 12/85 cases (14.12%) and from the ECA (type C) in 39/85 (45.88%) cases. The STA was missing bilaterally in 1/85 (1.18%) cases. Variants with bilateral asymmetry of the carotid origin of the STA were CCA/CB (type A/type B) in 8/85 (9.41%) cases, CCA/ECA (type A/type C) in 5/85 (5.88%) cases, CB/ECA (type B/type C) in 17/85 (20%) cases, and ECA/absent STA (type C/ABS) in 2/85 cases (2.35%).

Hyoid-related types 1–3 were classified by gender on the right side (Table A5), left side (Table A6), and overall on 170 sides. The infrahyoid type 1 of STA was found in 47.06% of cases. Hyoid type 2 was found in 20.59% (2a), 0.59% (2b), and 4.71% (2c). The suprahyoid type 3 was found in 21.18% (3a), 0% (3b), and 3.53% (3c) (Table A7).

The statistical tests demonstrated that hyoid-related types 1–3 of the STA were significantly influenced by the A-C types of the carotid origin of the STA (*p* < 0.05). Gender did not significantly influence the STA types we determined (*p* > 0.05).

### 3.2. The Unilateral Combinations of Variables

Unilateral combinations were determined in a total of 85 cases. On the right side, in the overall group (N = 85), we did not find the unilateral combinations of types A2b, A2c, A3a, A3b, A3c, B2b, B3b, B3c, C2b, and C3b. On the left side, we did not find the combinations of types A2b, A2c, A3b, A3c, B2b, B3b, B3c, and C3b. The A1 combination was found in 8/85 cases (4.82%). The A2a combination was found in 6/85 cases (3.61%). The A3a combination was found in 1/85 cases (0.6%). The B1 combination was found in 26/85 cases (15.66%). The B2a combination was found in 13/85 cases (7.83%). The B2c combination was found in 4/85 cases (2.41%). The B3a combination was found in 6/85 cases (3.61%). The C1 combination was found in 46/85 cases (27.71%) and was the prevailing combination. The C2a combination was found in 16/85 cases (9.64%). The C2b combination was found in 1/85 cases (0.6%). The C2c combination was found in 3/85 cases (1.81%). The C3a combination was found in 29/85 cases (17.47%), and the C3c combination was found in 7/85 cases (4.22%). The repartition of combinations in the overall group by sides is presented in Table A8.

**Figure 2:**
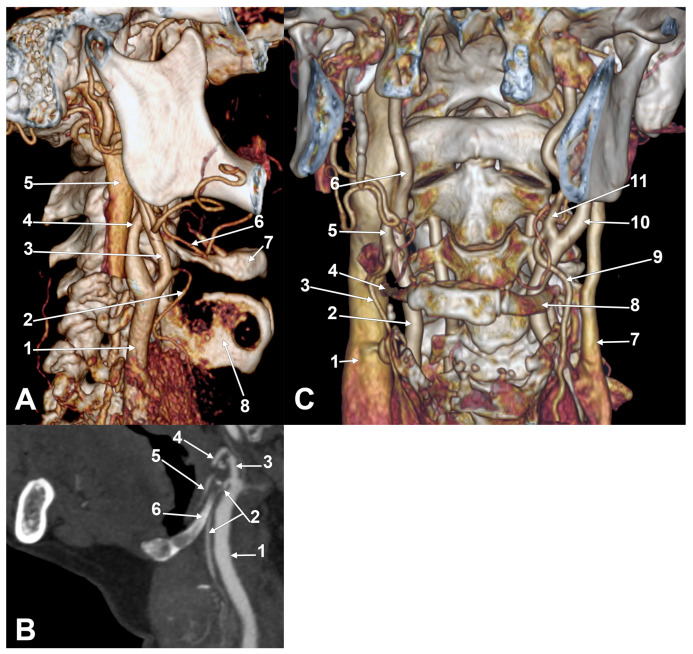
(**A**) Superior thyroid artery with infrahyoid origin from external carotid artery (unilateral C1 combination) and perilaryngeal tortuous course. Male case. Three-dimensional volume rendering. Right side. Lateral view. 1. Common carotid artery; 2. superior thyroid artery; 3. external carotid artery; 4. internal carotid artery; 5. internal jugular vein; 6. lingual artery; 7. body of hyoid; 8. thyroid cartilage. (**B**) Superior thyroid artery with origin at level of hyoid tubercle and initial course internal to greater hyoid horn (type C2b). Oblique sagittal section. Female case. 1. Common carotid artery; 2. superior thyroid artery; 3. external carotid artery; 4. facial artery; 5. lingual artery; 6. greater hyoid horn. (**C**) Bilateral association type XXXIII (C3a-C3a). Female case. Three-dimensional volume rendering; left anterior oblique view. 1. Right internal jugular vein; 2. right common carotid artery; 3. right superior thyroid artery; 4. right greater hyoid horn; 5. right external carotid artery; 6. right internal carotid artery; 7. left internal jugular vein; 8. left greater hyoid horn; 9. left superior thyroid artery; 10. left internal carotid artery; 11. left external carotid artery.

**Figure A1:**
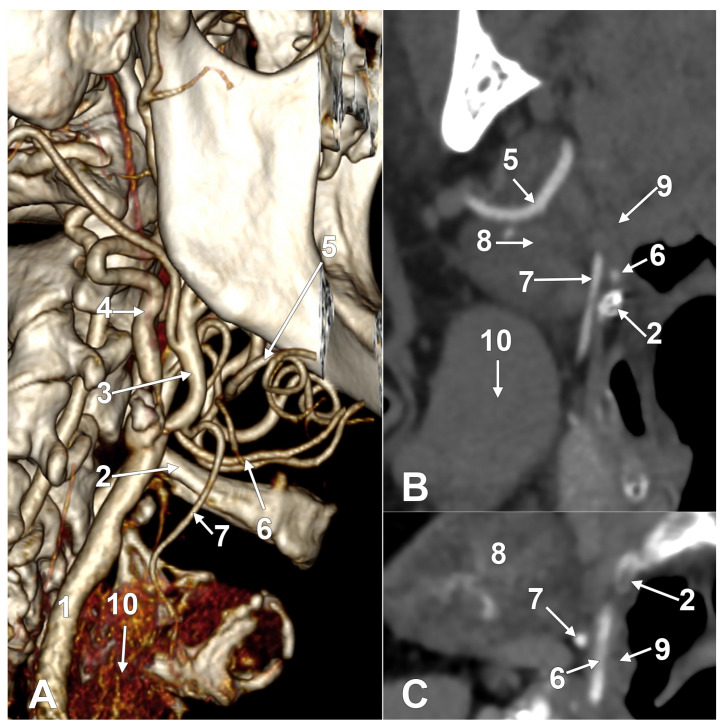
Right superior thyroid artery with suprahyoid origin and course over lingual artery and greater hyoid horn (C3a combination). Male case. (**A**) Three-dimensional volume rendering, right side, lateral view. (**B**) Coronal section through right greater hyoid horn, anterior view. (**C**) Axial section through right superior thyroid and lingual arteries, inferior view. 1. Common carotid artery; 2. greater hyoid horn; 3. external carotid artery; 4. internal carotid artery; 5. facial artery; 6. lingual artery; 7. superior thyroid artery; 8. submandibular gland; 9. hyoglossus muscle; 10. internal jugular vein.

Interestingly, on the right side prevailed combinations C1, C2a (Figure 1C), C3a (Figure 1D,E and Figure A1), and B1, while on the left side prevailed combinations C1 (Figure 2A), B1, B2a, and C3a. The combinations with <5% prevalence were regarded as rare. On the right side, such rare combinations were A1, A2a, B2a, B2c, C2c, B3a, and C3c. The rare combinations on the left side were A2a, B2c, C2a, C2b (Figure 2B), C2c, A3a, B3a, and C3c. The distribution of combination types by genders and sides is presented in Table A9. In a case with the C3a combination (Figure A1), the STA laterally crossed the lingual artery, which was coursing on the lateral side of the hyoglossus, superiorly to the GHHB.

### 3.3. The Bilateral Associations of the Unilateral Combinations of Variables

**Figure A2:**
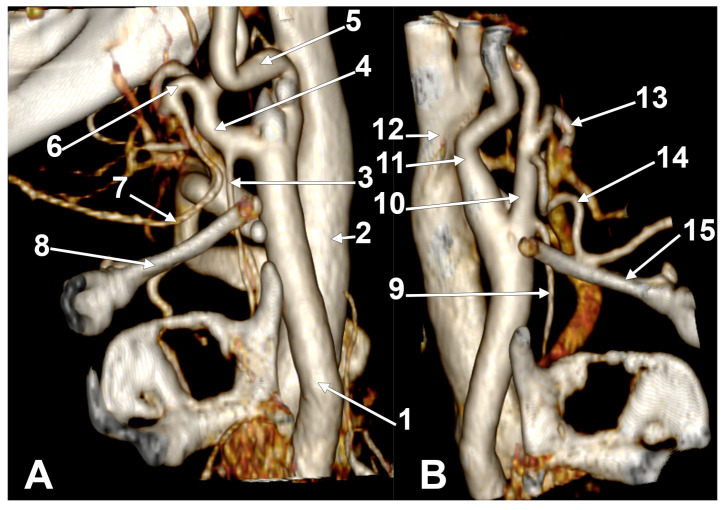
Superior thyroid arteries with suprahyoid origin from external carotid arteries and course over greater hyoid horn (bilateral association type XXXIII—C3a-C3a). Male case. Three-dimensional volume renderings. (**A**) Right side, antero-medial view. (**B**) Left side, medial view. 1. Right common carotid artery; 2. right internal jugular vein; 3. right superior thyroid artery; 4. right external carotid artery; 5. right internal carotid artery; 6. linguofacial trunk; 7. right lingual artery; 8. greater hyoid horn; 9. left superior thyroid artery; 10. left external carotid artery; 11. left internal carotid artery; 12. left internal jugular vein.

The bilateral associations of unilateral combinations of STA types were classified into thirty-seven types (I–XXXVII) (Table A10). The typical bilateral association symmetrical type XVIII (C1-C1) was found in only 13/85 cases (15.29%), indicating a bilateral infrahyoid origin of the STA from the ECA in 13 cases (15.29%). In 9/85 cases (10.58%), we found the type XXXIII association (C3a-C3a) and bilateral symmetry for the suprahyoid origin of the STA from the ECA, with the STA descending over the GHHB (Figure 2C and Figure A2). In 8/85 cases (9.41%), we found the XV asymmetric association C1-B1—infrahyoid origins of the STA from the ECA and CB, respectively.

**Figure 3:**
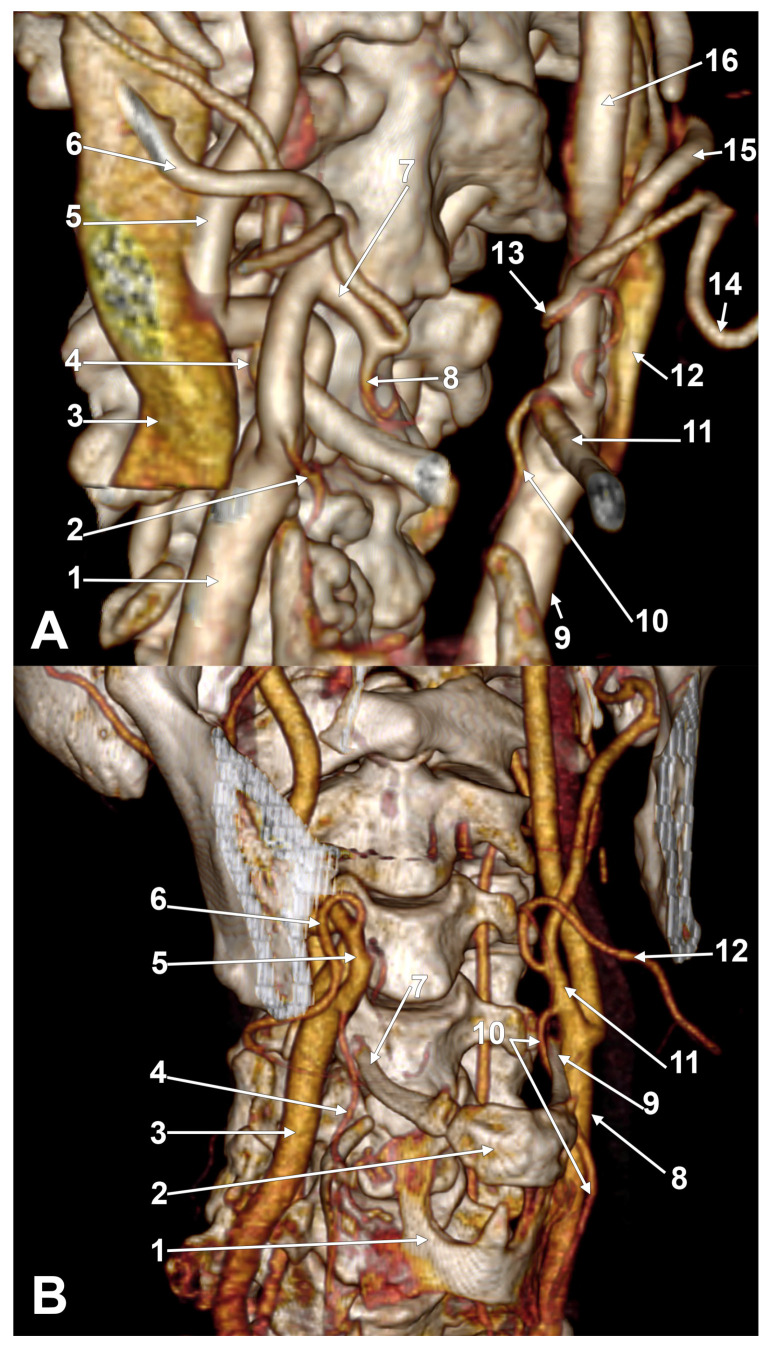
(**A**) Superior thyroid arteries originating from external carotid arteries at asymmetric hyoid levels: immediately infrahyoid (type 1) on right side and hyoid (type 2c) on left side. Bilateral association C1-C2c (type XIX), female case. Three-dimensional volume rendering. Right antero-infero-lateral view. 1. Right common carotid artery; 2. right superior thyroid artery; 3. right internal jugular vein; 4. right hyoid tubercle; 5. right internal carotid artery; 6. right external carotid artery; 7. right linguofacial trunk; 8. right lingual artery; 9. left common carotid artery; 10. left superior thyroid artery; 11. left greater hyoid horn; 12. left internal jugular vein; 13. left lingual artery; 14. left facial artery; 15. left external carotid artery; 16. left internal carotid artery. (**B**) Superior thyroid arteries with suprahyoid origin from external carotid arteries (types C3a on right side and C3c on left side, asymmetric association XXXIV). Overlapped hyoid bone and thyroid cartilage. Male case. AngioCT, three-dimensional volume rendering. Right anterolateral view. 1. Thyroid cartilage; 2. body of hyoid bone; 3. right common carotid artery; 4. right superior thyroid artery; 5. right external carotid artery; 6. right facial artery; 7. right greater horn of the hyoid; 8. left common carotid artery; 9. left greater horn of the hyoid; 10. left superior thyroid artery; 11. left external carotid artery; 12. left facial artery.

The cases with asymmetrical bilateral associations of unilateral combinations of types prevailed (Figure 3). A lowered hyoid bone overlapping the thyroid cartilage was found in one of these cases (Figure 3B). In the overall batch (N = 85), there were 33 (38.82%) symmetrical and 52 (61.18%) asymmetrical bilateral associations of unilateral combinations of types. In males (N_M_ = 53), there were 19 (35.85%) symmetrical and 34 (64.15%) asymmetrical associations. In females (N_F_ = 32), 14 (43.75%) symmetrical and 18 (56.25%) asymmetrical associations were found.

**Figure 4:**
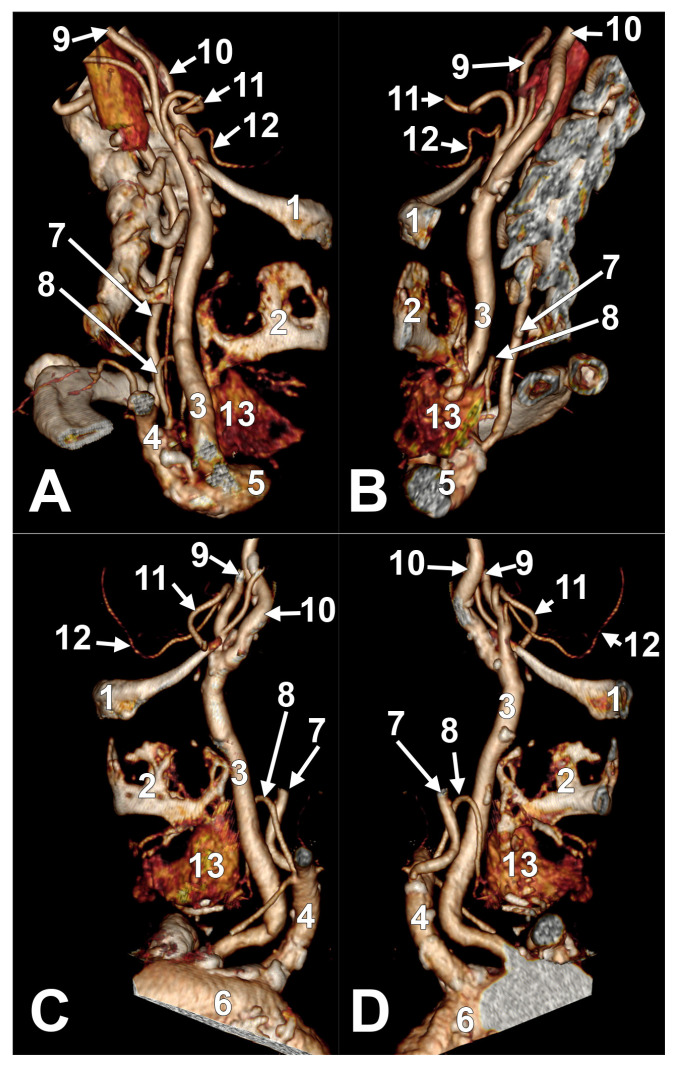
Bilateral absence of superior thyroid artery (type V association). Three-dimensional volume renderings. Right side, antero-lateral view (**A**) and antero-medial view (**B**). Left side, antero-inferior-lateral view (**C**) and infero-medial view (**D**). 1. Body of hyoid bone; 2. thyroid cartilage; 3. common carotid artery; 4. subclavian artery; 5. brachiocephalic trunk; 6. aortic arch; 7. vertebral artery; 8. inferior thyroid artery; 9. external carotid artery; 10. internal carotid artery; 11. facial artery; 12. lingual artery; 13. thyroid lobe.

**Figure 5:**
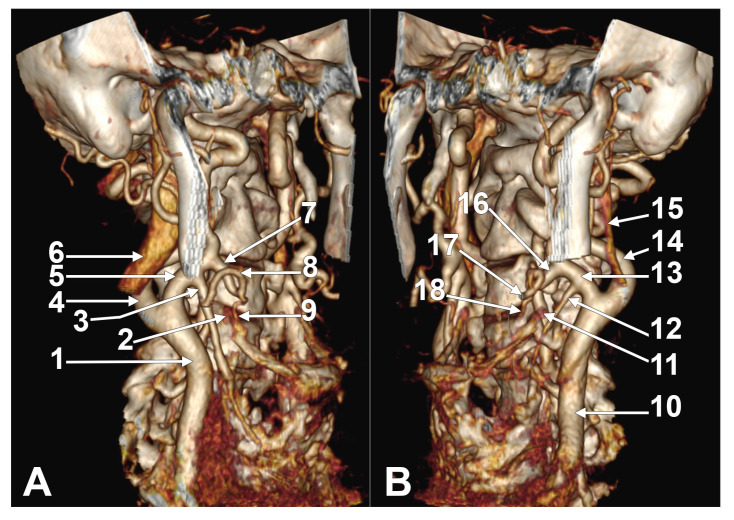
Superior thyroid arteries with suprahyoid origin from external carotid arteries (bilateral C3c type, bilateral symmetrical association type XXXVII). Bilateral linguofacial trunk. Three-dimensional volume renderings. (**A**) Right side, antero-lateral view. (**B**) Left side, antero-lateral view. 1. Right common carotid artery; 2. right hyoid tubercle; 3. right superior thyroid artery; 4. right internal carotid artery; 5. right external carotid artery; 6. right internal jugular vein; 7. right linguofacial trunk; 8. facial artery; 9. lingual artery; 10. left common carotid artery; 11. left hyoid tubercle; 12. left superior thyroid artery; 13. left external carotid artery; 14. left internal carotid artery; 15. left internal jugular vein; 16. left linguofacial trunk; 17. left facial artery; 18. left lingual artery.

Symmetrical bilateral associations were found for types III (A2a-A2a); V—bilaterally absent STA (Figure 4); VII (B1-B1); X (B2a-B2a); XVIII (C1-C1); XXIII (C2a-C2a); XXXIII (C3a-C3a); and XXXVII (C3c-C3c) (Figure 5, Table A11). The gender repartition of cases with asymmetrical bilateral associations is presented in Table A12.

## 4. Discussion

Previous anatomical studies of the STA did not investigate its hyoid-related variability [11,12,17,19,20,21,22], unlike the present study. Alagöz et al. (2005), cited by Anagnostopoulou and Mavridis (2014), found the origin of the STA from the ECA at the level of the GHHB, between 1.5 cm above and 1 cm below it [19,23]. They did not split the hyoid-related variants as we did. Won (2016) studied 30 cadavers and identified only the infrahyoid origin of the STA, 0.9 ± 0.4 mm below the hyoid bone [24]. In the present study, we identified STAs of suprahyoid, hyoid, or infrahyoid origin. In a recent meta-analysis, several studies [25,26,27,28,29,30,31] were documented that analysed the relationship of the STA to the upper thyroid cartilage margin [16]. The origin of the STA was 66.14% superior to, 15.08% at, and 13.34% inferior to the superior margin of the thyroid cartilage [16]. The hyoid bone was not considered a topographic landmark for the STA [25,26,27,28,29,30,31].

Lo et al. (2006) found that the STA originated from the CB in 52% of 36 cadavers, from the ECA in 46.2%, and from the CCA in 1.5% [1]. They discussed the possibility that the origin of the STA from the CCA may be more frequent than suggested in the literature. However, in the 85 angioCT cases we studied, we found that the STA originated from the CCA in 8.82%, from the CB in 28.82%, and from the ECA in 60%; therefore, there is a lower prevalence of the CB origin of the STA and a higher prevalence of CCA and ECA origins of the STA. Al-Rafiah et al. (2011) found that the STA originated from the ECA in 80% of 30 cadavers [6]. The use of various methods and study samples of varying sizes may explain the prevalence of different origin sites of the STA from the carotid axis in different studies, including ours.

In major surgery, in the lateral compartment of the neck, the STA must be identified to avoid a life-threatening injury [32]. It is crucial to ligate the STA close to its origin during thyroid surgery to avoid damage to the superior laryngeal nerve [33]. This careful ligation helps prevent complications such as superior laryngeal nerve palsy, which can occur if the nerve is inadvertently injured during the procedure [33]. However, the point of origin of the STA has always been debated [32]. The controversies are based on heterogeneous results of published studies [32]. The CB and ECA are not the only possible origins of the STA [32]. In many cases, the STA originates directly from the CCA [32]. In addition, there are reports in which the STA either originates from the ICA or is absent [32]. Poutoglidis et al. (2023) documented a systematic review of 5488 specimens [32]. The authors found the ECA as the most common site of STA origin (55%), followed by the CB (27.5%) and the CCA (15%) [32]. The STA origin from the ICA had a prevalence of 0.055% [32]. A thyro-occipito-pharyngeal common trunk of the STA and occipital and ascending pharyngeal arteries was also reported [14]. The STA may rarely form common trunks with other anterior branches of the ECA, such as the TLT and TLFT [10,16]. We did not find such common trunks in the batch we used. The retropharyngeal course of the STA (posterior to the piriform sinus) was recently reported [34]. Using an operating microscope to identify arteries might help prevent injuries [4].

Different authors studied the same types of origin of the STA from the carotid axis but used different classification systems. Vazquez et al. (2009) traced the origin of the STA in 165 dissected cadavers [12]. They classified it into four types: type I, originating from the CB; type II, originating from the CCA; type III, originating from the ECA; and type IV, originating from common trunks, TLT, or TLFT [12]. Ongeti and Ogeng’o (2012) studied the anatomical variations in the STA in 46 cadavers by dissection and used a classification system similar to that used by Vazquez et al. (2009) [12,21]. All the STAs with origins from the CCA were associated with high CB [21]. The authors found the bilateral asymmetry of the STA’s origin in the carotid system in 6.5% of cases [21]. The present study found the bilateral asymmetry of the STA’s origin in 37.64% of cases. Ozgur et al. (2009) studied 20 cadavers and categorised the origin of STA with respect to the CB as follows: superior to the CB (25%), inferior to the CB (35%), and at the same level with it (40%) [22]. These authors also looked at the branching pattern of the STA [22]. Natsis et al. (2011) studied 100 human cadavers. They proposed a classification of the anatomical types of the anterior branches of the ECA as follows: type I—STA with separate origin, either from the CCA, CB, or ECA; type II—common trunks [11]. Gupta et al. (2014) studied 25 angiographs from 15 cases [20]. On the right side, the STA originated from the ECA (71.5%), CB (21.5%), or the CCA (7%) [20]. On the left, the STA arose from the ECA (72.5%), CB (18.5%), or ICA (9%) [20]. They also defined three branching patterns of the STA [20]. Anagnostopoulou and Mavridis (2014) studied the STA by dissection in 68 cadavers [19]. They classified the STA’s origin into three types: type A, originating from the CCA; type B, originating from the ECA, either directly or via common trunks; and type C, originating from the CB [19]. In the present study, we preferred to list these types in anatomical sequence: originating from the CCA (type A), originating from the CB (type B), and originating from the ECA (type C).

Different anatomical studies, conducted using various methodologies, have consistently identified a relatively constant set of anatomical variables of the STA, as demonstrated by several meta-analyses on the STA [16,35,36]. Triantafyllou et al. (2024) considered the origin of the STA distinctly from the CCA and the CB in addition to the typical variant, the origin of the STA from the ECA [16]. However, these authors also considered the STA’s origin from common trunks, specifically TLT or TLFT. They determined the site of origin of these trunks to be either the ECA or the CCA [16]. We present the pooled prevalence of the STA’s origin, as determined by Triantafyllou et al. (2024), including that for the common trunks, in Table 1.

A meta-analysis by Toni et al. (2003) calculated an almost 100% prevalence of the STA overall and of each side in both Caucasians and Asians [36]. According to that meta-analysis, the STA was missing in Caucasians in 1% of cases from the left side [36]. In our batch, the STA was absent in one case on the right side (0.59%) and in three cases (1.76%) on the left side. Thus, the absence of the STA seems to have a predilection for the left side of the neck.

Toni et al. (2003) determined that the STA originating from the ECA (type C in our study) had the highest prevalence in both meta-analysed anthropological groups, Caucasians and Asians [36]. The authors also determined that in Asians, the STA originating from the CCA was more prominent than in Caucasians [36]. This origin corresponds to types A and B in our study because Toni et al. (2003) recorded the STA originating from the CB as an origin from the CCA as both have a common embryological origin from the third branchial arch and the CB can be anatomically considered the highest portion of the CCA [36]. For this reason, in our Caucasian batch, the STA originating from both the CCA and CB was detected in 37.65%, and that from the ECA was detected in 60% of cases with present STA. The ECA origin of the STA was more common on the right side, and the CCA origin was more common on the left side for Caucasians and Asians [35,36]. In our study, the STA originating from the ECA on the right side had a prevalence of 36.47% and only 23.53% on the left side.

Toni et al. (2003) had no data on bilateral symmetry in Caucasians. Still, in Asians, the STA origin was symmetric in about half of the cases, with symmetric origin from the ECA being more frequent than symmetric origin from the CCA [36]. In the present study, we observed bilaterally symmetric A–C types in 53/85 cases (62.35%), with symmetric origin from the ECA being found in 45.88% of cases and symmetric origin from the CCA and CB in 15.3% of cases. This finding is in agreement with the analysis conducted by these authors. However, our study also examined the relationship between the STA and the hyoid. We obtained 37 patterns of bilateral association of unilateral combination types, a more complex result than the symmetry/asymmetry of the STA origin types in the carotid axis. An error in identifying the bilateral asymmetric origin of the STA may reduce the effectiveness of the bilateral cannulation of the STA [35].

In the 85 cases studied here, we found 1 case with bilaterally absent STA (1.18%) and 2 with a unilaterally absent STA (2.35%). This is a higher prevalence compared to other studies. Poutoglidis et al. (2023) published a systematic review of the STA and established that its absence occurs in 0.45% [32]. Herrera-Nunez et al. (2020) found the unilateral absence of the STA in 1.3% of 76 cases (152 carotid axes) examined by angioCT [37]. Moriggl and Sturm (1996) reported one case with an STA and both inferior thyroid arteries absent; the remaining STA originated from the CCA [38]. Esen et al. (2018) studied 640 cases explored by angioCT [39]. In 0.9% of cases on the right and 1.9% on the left, the STA was not identified [39]. In 0.3% of cases, the STA was absent bilaterally [39]. An absent STA makes the inferior thyroid artery, in the absence of the thyroid ima artery, a single arterial pedicle of the thyroid lobe. This must be taken into account during surgery.

Mehta et al. (2010) reported a dissected case with a so-called absent right superior thyroid artery (STA); the authors presented the dissection in a left anterolateral view, identifying the thyroid isthmus as the right thyroid lobe, and in the dissection, thyroid arterial branches were seen on the right, but not their origin [40]. It is quite possible that the absence of the STA was simply an anatomical misinterpretation.

Researchers must be careful when indicating the absence of the STA in imaging studies as finer arteries may be misidentified as absent during the image analysis process [16]. This observation is emphasised by the subgroup analysis of the Greek group, which showed an estimate of 0.00% regarding the STA’s absence in cadaver studies and an estimate of 0.84% in imaging studies [16].

In one case with the C2a type of STA, the STA crossed over the lateral side of the lingual artery, which, in turn, had a lateral trajectory of the hyoglossus muscle above the GHHB. This direct relationship of the STA and the lingual artery which we found here is not reported in the literature and appears as a novelty.

In one case with bilateral C2a-C2a association, thus showing hyoid origins of the STA, we identified that the hyoid bone collapsed over the thyroid cartilage of the larynx. This possibility was recently indicated a 3% prevalence [41]. Such an anatomical possibility alters the conventional cervical landmarks [41].

Mechanical compression of the carotid arteries may lead to flow changes [42]. We consider, however, that a possible mechanical compression of an STA directly related to the hyoid bone could not be followed by significant thyroid vascular deficits because bilateral anastomoses of the thyroid arteries can play a compensatory role. It is considered, however, that ischaemia of thyroid tissue could lead to hypothyroidism [43].

The STA is a surgical landmark for identifying the external branch of the superior laryngeal nerve [17]. This fits with a typical infrahyoid origin of the STA. However, we found suprahyoid origins of the STAs that further coursed over the GHHB. It is reasonable to consider that in such cases, the external branch of the superior laryngeal nerve may not follow the course of the STA. Therefore, an STA with an aberrant course loses its value for the surgical identification of that nerve.

The STA has essential roles in head and neck surgery [17]. It is commonly used as a recipient vessel in microvascularised free tissue grafts, for selective thyroid and other head and neck tumour embolisation, emergency cricothyroidotomy, radical neck dissection, diagnostic and therapeutic catheterisation, plastic surgery, aneurysm reconstruction, and carotid endarterectomy [17]. It has a significant role in thyroid gland surgery [17].

The accurate identification of the STA is also very important during the assessment of peak systolic velocity using Doppler ultrasound [44]. Elevated values are characteristic of Graves’ disease [44]. In destructive thyroiditis, these values are significantly lower [44].

Our study brought into focus the potential bilateral topographical asymmetry of the STA. This is a novel finding. Numerous previous studies of the STA were based on cadaver dissections and focused solely on the variable origin of the STA from the carotid axis [44]. We consider that an anatomical study using angioCT files is more reliable because the findings could be adequately verified on planar slices and demonstrated by three-dimensional volume renderings.

### 4.1. The Limitations of the Study

The present study was designed as an anatomo-topographical one. It did not aim to select and observe distinctive lots of young and older adult patients. Neither the tortuosities of the main carotid arteries were recorded. Dilated and tortuous carotid arteries are more common in older subjects compared to younger ones [45].

The position of the hyoid bone has been described in conjunction with changes in tongue, mandible, and head positions [46]. A statistically weak negative correlation was found between the age and the hyoid bone area [47]. This could not influence the topography of the STA. The location of the hyoid bone was found at the C3 vertebral level in 58.0% of males and 80.0% of females. The C3 rate was statistically higher in females [47]. We did not record the vertebral levels of the STA origin in the present study. When the effect of ageing on the anatomical position of the thyroid gland was investigated in 122 patients, it was found that the position of the gland did not change significantly relative to anatomic landmarks in the head or neck [48]. However, the position of the thyroid gland was dependent on changes in the cervical spine height, the distance from the hyoid bone to the hard palate, and the tracheal angle [48]. Therefore, recording the vertebral level of the STA origin should have been correlated with the patients’ ages.

### 4.2. Future Directions

Aristokleous et al. (2011) found that head rotation causes significant variations in the CB’s angle and the ICA’s angle, a slight increase in planarity and asymmetry angles for both CCAs, minor and variable curvature changes for the CCA and its branches, slight tortuosity changes for the branches but not for the CCA, and unsubstantial alterations in area and diameter ratios [49]. However, although the geometry of the CB and its branches may vary during head rotation, this may not significantly modify the origin sites of the ECA’s branches compared to the hyoid. During angioCT scans, patients are placed in a supine position; therefore, head rotation cannot be implicated in the variable topographic patterns of any artery. However, further studies are warranted to investigate additional factors that may influence the topography of the STA. Specifically, an analysis of the potential correlation between the STA and the cervical (C2–C7) Cobb angle may yield novel insights into how cervical spine alignment affects vascular morphology and topography. Similarly, the impact of body mass index (BMI) on STA’s topography may dictate whether patient-specific anthropometric factors play any role in vascular variation. These factors may translate into more individualised neck surgery and risk assessment approaches.

## 5. Conclusions

In conclusion, the carotid origin of the STA is predictable, but its relations with the hyoid bone are variable. Bilateral associations of combined variants of the STA are unpredictable. The types of the carotid origin of the STA significantly influence the hyoid-related types of the STA. Gender does not significantly influence these types.

## Figures and Tables

**Table 1:** The pooled prevalence of variants of origin of the superior thyroid artery (STA) as determined by Triantafyllou et al. (2024) [16]. CCA: common carotid artery, CB: carotid bifurcation, ECA: external carotid artery, ICA: internal carotid artery, TLT: thyrolingual trunk, TLFT: thyrolinguofacial trunk.

Origin of STA	Pooled Prevalence
CCA	13.09%
CB	25.36%
ECA	56.94%
ICA	0.00% (2 cases)
TLT from the CCA	0.01%
TLT from the CB	0.00% (11 cases)
TLT from the ECA	0.61%
TLFT from the CB	0.00% (2 cases)
TLFT from the ECA	0.09%
Absent STA	0.09%

## Data Availability

The datasets used and analysed during the current study are available from the corresponding author upon reasonable request.

## Abbreviations

The following abbreviations are used in this manuscript:

ECA	external carotid artery
GHHB	greater horn of hyoid bone
ICA	internal carotid artery
CCA	common carotid artery
CB	carotid bifurcation
STA	superior thyroid artery
TLT	thyrolingual trunk
TLFT	thyrolinguofacial trunk

## Appendix A

### Appendix A.1. Tables

**Table A1:** Classification systems used for superior thyroid artery assessment.

Parameter	Classification	Description
STA origin type	A	Origin from the CCA
	B	Origin from the CB
	C	Origin from the ECA
	D	Origin from the ICA
	E	Origin from the TLT
	F	Origin from the TLFT or other rare variants
Topographic origin relative to GHHB	Type 1	Origin inferior to the GHHB
	Type 2	Origin at the level of the GHHB
	2a	Course lateral to the GHHB
	2b	Course medial to the GHHB
	2c	Course posterior to the GHHB
	Type 3	Origin superior to the GHHB
	3a	Course lateral to the GHHB
	3b	Course medial to the GHHB
	3c	Course posterior to the GHHB

**Table A2:** Unilateral combinations of types.

Unilateral Combinations of Types	Carotid Origin of STA (Types A–C)	Hyoid Level of Origin and Course of STA (Types 1–3)
A1	CCA	infrahyoid (type 1)
B1	CB	infrahyoid (type 1)
C1	ECA	infrahyoid (type 1)
A2a	CCA	hyoid, lateral to the GHHB
A2b	CCA	hyoid, medial to the GHHB
A2c	CCA	hyoid, posterior to the GHHB
B2a	CB	hyoid, lateral to the GHHB
B2b	CB	hyoid, medial to the GHHB
B2c	CB	hyoid, posterior to the GHHB
C2a	ECA	hyoid, lateral to the GHHB
C2b	ECA	hyoid, medial to the GHHB
C2c	ECA	hyoid, posterior to the GHHB
A3a	CCA	suprahyoid, lateral to the GHHB
A3b	CCA	suprahyoid, medial to the GHHB
A3c	CCA	suprahyoid, posterior to the GHHB
B3a	CB	suprahyoid, lateral to the GHHB
B3b	CB	suprahyoid, medial to the GHHB
B3c	CB	suprahyoid, posterior to the GHHB
C3a	ECA	suprahyoid, lateral to the GHHB
C3b	ECA	suprahyoid, medial to the GHHB
C3c	ECA	suprahyoid, posterior to the GHHB

**Table A3:** Origins of superior thyroid arteries (types A–C) in investigated lot (N = 170 sides) by gender (count and prevalence). M, males; F, females; ABS, absent.

	Type A	Type B	Type C	ABS
M, 106 sides	9 8.49%	38 35.85%	56 52.83%	3 2.83%
F, 64 sides	6 9.38%	11 17.19%	46 71.88%	1 1.56%
TOTAL, 170 sides	15 8.82%	49 28.82%	102 60%	4 2.35%

**Table A4:** Origins of superior thyroid arteries (types A-C) in investigated lot (N = 170 sides) by sides and genders (count and prevalence). M, males; F, females; ABS, absent.

	Type A Right	Type A Left	Type B Right	Type B Left	Type C Right	Type C Left	ABS Right	ABS Left
M, 106 sides	3 2.83%	6 5.66%	14 13.21%	24 22.64%	35 33.02%	21 19.81%	1 0.94%	2 1.89%
F, 64 sides	1 1.56%	5 7.81%	4 6.25%	7 10.94%	27 42.19%	19 29.69%	0 0%	1 1.56%
TOTAL, 170 sides	4 2.35%	11 6.47%	18 10.59%	31 18.24%	62 36.47%	40 23.53%	1 0.59%	3 1.76%

**Table A5:** Hyoid-related origin and course of superior thyroid arteries (types 1–3) by gender on right side (count, prevalence). ABS, absent superior thyroid arteries.

	Type 1	Type 2a	Type 2b	Type 2c	Type 3a	Type 3b	Type 3c	ABS
M (53)	25 47.17%	9 16.98%	0 0%	1 1.89%	16 30.19%	0 0%	1 1.89%	1 1.89%
F (32)	13 40.63%	8 25%	0 0%	2 6.25%	7 21.88%	0 0%	2 6.25%	0 0%
TOTAL (85)	38 44.71%	17 20%	0 0%	3 3.53%	23 27.06%	0 0%	3 3.53%	1 1.18%

**Table A6:** Hyoid-related origin and course of superior thyroid arteries (types 1–3) by gender on left side (count, prevalence). ABS, absent superior thyroid arteries.

	Type 1	Type 2a	Type 2b	Type 2c	Type 3a	Type 3b	Type 3c	ABS
M (53)	25 48.08%	12 23.08%	0 0%	2 3.85%	9 17.31%	0 0%	2 3.85%	2 3.85%
F (32)	17 53.13%	6 18.75%	1 3.13%	2 6.25%	4 12.5%	0 0%	1 3.13%	1 3.13%
TOTAL (85)	42 50%	18 21.43%	1 1.19%	4 4.76%	13 15.48%	0 0%	3 3.57%	3 3.57%

**Table A7:** Hyoid-related origin and course of superior thyroid arteries (types 1–3) by gender on both sides (count, prevalence). ABS, absent superior thyroid arteries.

	Type 1	Type 2a	Type 2b	Type 2c	Type 3a	Type 3b	Type 3c	ABS
M (106)	50 47.17%	21 19.81%	0 0%	4 3.77%	25 23.58%	0 0%	3 2.83%	3 2.83%
F (64)	30 46.88%	14 21.88%	1 1.56%	4 6.25%	11 17.19%	0 0%	3 4.69%	1 1.56%
TOTAL (170)	80 47.06%	35 20.59%	1 0.59%	8 4.71%	36 21.18%	0 0%	6 3.53%	4 2.35%

**Table A8:** Unilateral combinations of types in overall lot (N = 85).

Unilateral Combinations of Types	Right Side (Count, %)	Left Side (Count, %)
A1	2, 2.35%	6, 7.06%
B1	10, 11.76%	16, 18.82%
C1	26, 30.59%	20, 23.53%
A2a	2, 2.35%	4, 4.71%
A2b		
A2c		
B2a	3, 3.53%	10, 11.76%
B2b		
B2c	2, 2.35%	2, 2.35%
C2a	12, 14.12%	4, 4.71%
C2b		1, 1.18%
C2c	1, 1.18%	2, 2.35%
A3a		1, 1.18%
A3b		
A3c		
B3a	3, 3.53%	3, 3.53%
B3b		
B3c		
C3a	20, 23.53%	9, 10.59%
C3b		
C3c	3, 3.53%	4, 4.71%

**Table A9:** Unilateral combinations of types by gender (N_M_ = 53; N_F_ = 32).

Unilateral Combinations of Types	Males		Females	
	Right Side (Count, %)	Left Side (Count, %)	Right Side (Count, %)	Left Side (Count, %)
A1	2, 3.77%	3, 5.66%		3, 9.38%
B1	8, 15.09%	12, 22.64%	2, 6.25%	4, 12.5%
C1	15, 28.3%	10, 18.87%	11, 34.38%	10, 31.25%
A2a	1, 1.89%	2, 3.77%	1, 3.13%	2, 6.25%
A2b				
A2c				
B2a	2, 3.77%	7, 13.21%	1, 3.13%	3, 9.38%
B2b				
B2c	1, 1.89%	2, 3.77%	1, 3.13%	
C2a	6, 11.32%	3, 5.66%	6, 18.75%	1, 3.13%
C2b				1, 3.13%
C2c			1, 3.13%	2, 6.25%
A3a		1, 1.89%		
A3b				
A3c				
B3a	3, 5.66%	3, 5.66%		
B3b				
B3c				
C3a	13, 24.53%	5, 9.43%	7, 21.88%	4, 12.5%
C3b				
C3c	1, 1.89%	3, 5.66%	2, 6.25%	1, 3.13%

**Table A10:** Bilateral associations of unilateral combinations of types (N = 85). AMS, absent.

Type of Bilateral Association	Associated Unilateral Combinations	Count, %	Type of Bilateral Association	Associated Unilateral Combinations	Count, %
I	B1-A1	4, 4.7%	XX	C2a-A2a	2, 2.35%
II	C1-A1	3, 3.53%	XXI	C2a-B2a	1, 1.17%
III	A2a-A2a	1, 1.17%	XXII	C2a-C1	4, 4.7%
IV	A2a-B2a	1, 1.17%	XXIII	C2a-C2a	3, 3.53%
V	ABS-ABS	1, 1.17%	XXIV	C2a-C2c	1, 1.17%
VI	B1-A2a	1, 1.17%	XXV	C2a-C3c	1, 1.17%
VII	B1-B1	3, 3.53%	XXVI	C2c-C2b	1, 1.17%
VIII	B2a-B1	2, 2.35%	XXVII	C3a-ABS	2, 2.35%
IX	B1-B2c	2, 2.35%	XXVIII	C3a-B1	2, 2.35%
X	B2a-B2a	2, 2.35%	XXIX	C3a-B2a	2, 2.35%
XI	B2c-A1	1, 1.17%	XXX	C3a-B3a	1, 1.17%
XII	B2c-B3a	1, 1.17%	XXXI	C3a-C1	1, 1.17%
XIII	B3a-A3a	1, 1.17%	XXXII	C3a-C2a	1, 1.17%
XIV	B3a-B2a	2, 2.35%	XXXIII	C3a-C3a	9, 10.58%
XV	C1-B1	8, 9.41%	XXXIV	C3a-C3c	2, 2.35%
XVI	C1-B2	1, 1.17%	XXXV	C3c-B1	1, 1.17%
XVII	C1-B3a	1, 1.17%	XXXVI	C3c-C1	1, 1.17%
XVIII	C1-C1	13, 15.29%	XXXVII	C3c-C3c	1, 1.17%
XIX	C1-C2c	1, 1.17%			

**Table A11:** Cases with bilaterally symmetrical associations of combinations by gender (count) and in overall lot (count, prevalence). M, males; F, females.

Gender/Type	Type III	Type V	Type VII	Type X	Type XVIII	Type XXIII	Type XXXIII	Type XXXVII
M = 53	1	1	3	1	6	2	5	0
F = 32	0	0	0	1	7	1	4	1
TOTAL	1 (1.17%)	1 (1.17%)	3 (3.52%)	2 (2.35%)	13 (15.29%)	3 (3.52%)	9 (10.58%)	1 (1.17%)

**Table A12:** Asymmetrical bilateral associations by gender (count). M, males; F, females.

Bilateral Associations	Types	M	F	Bilateral Associations	Types	M	F
B1-A1	I	3	1	C2a-B2a	XXI	1	0
C1-A1	II	2	1	C2a-C1	XXII	1	3
A2a-B2a	IV	0	1	C2a-C2c	XXIV	0	1
B1-A2a	VI	0	1	C2a-C3c	XXV	1	0
B2a-B1	VIII	2	0	C2c-C2b	XXVI	0	1
B1-B2c	IX	2	0	C3a-ABS	XXVII	1	1
B2c-A1	XI	0	1	C3a-B1	XXVIII	1	1
B2c-B3a	XII	1	0	C3a-B2a	XXIX	1	1
B3a-A3a	XIII	1	0	C3a-B3a	XXX	1	0
B3a-B2a	XIV	2	0	C3a-C1	XXXI	1	0
C1-B1	XV	6	2	C3a-C2a	XXXII	1	0
C1-B2	XVI	1	0	C3a-C3c	XXXIV	2	0
C1-B3a	XVII	1	0	C3c-B1	XXXV	0	1
C1-C2c	XIX	1	1	C3c-C1	XXXVI	1	0
C2a-A2a	XX	1	1				
